# Representation of the Universe as a Dendrogramic Hologram Endowed with Relational Interpretation

**DOI:** 10.3390/e23050584

**Published:** 2021-05-08

**Authors:** Oded Shor, Felix Benninger, Andrei Khrennikov

**Affiliations:** 1 Felsenstein Medical Research Center, Beilinson Hospital, Petach Tikva 49100, Israel; shor.oded@gmail.com (O.S.); benninger@tauex.tau.ac.il (F.B.); 2 Sackler Faculty of Medicine, Tel Aviv University, Tel Aviv 6997801, Israel; 3 Department of Neurology, Rabin Medical Center, Petach Tikva 4941492, Israel; 4 Faculty of Technology, Department of Mathematics, Linnaeus University, 351 95 Växjö, Sweden

**Keywords:** realist interpretation, classical vs. quantum: implicate vs. explicate order, p-adic numbers, dendrograms, Leibniz principle of the Identity of Indiscernible, holographic principle, Rovelli’s relational quantum mechanics, Smolin’s dynamics of difference, measurement problem, Bell correlations

## Abstract

A proposal for a fundamental theory is described in which classical and quantum physics as a representation of the universe as a gigantic dendrogram are unified. The latter is the explicate order structure corresponding to the purely number-theoretical implicate order structure given by p-adic numbers. This number field was zero-dimensional, totally disconnected, and disordered. Physical systems (such as electrons, photons) are sub-dendrograms of the universal dendrogram. Measurement process is described as interactions among dendrograms; in particular, quantum measurement problems can be resolved using this process. The theory is realistic, but realism is expressed via the the Leibniz principle of the *Identity of Indiscernibles*. The classical-quantum interplay is based on the degree of indistinguishability between dendrograms (in which the ergodicity assumption is removed). Depending on this degree, some physical quantities behave more or less in a quantum manner (versus classic manner). Conceptually, our theory is very close to Smolin’s dynamics of difference and Rovelli’s relational quantum mechanics. The presence of classical behavior in nature implies a finiteness of the Universe-dendrogram. (Infinite Universe is considered to be purely quantum.) Reconstruction of events in a four-dimensional space type is based on the holographic principle. Our model reproduces Bell-type correlations in the dendrogramic framework. By adjusting dendrogram complexity, violation of the Bell inequality can be made larger or smaller.

## 1. Introduction

The last several years have been characterized by new attempts to analyze quantum mechanics from a realist perspective [1,2,3,4]. Our theory, the *Dendrographic Hologram Representation of Physical Processes* (DH theory), originates from the very natural foundational principle, namely, Leibniz’s principle of the identity of the indiscernible [5,6]. The consequences of endorsing this principle (or rather declining the Leibniz principle of the assumption of identity in the micro/quantum world) are the rejection of ergodicity which is central in all of the current physical formulation [7]. In turn, this process will lead to the formulation of implicate and explicate orders and the holographic principle [8], which comes about very naturally. Moreover, we then come to the conclusion and understanding of the measurement problem as a simple apparent dynamic in which classicality suggests a bound universe. We note that Barbour’s and Smolin’s maximal variety paradigm [9] is a consequence of our results on the measurement process/apparent dynamics.

The DH-theory is closely related to Bohm’s paradigm [8] of the implicate versus explicate orders and the holographic principle, which plays the main role in string theory and quantum gravity [10,11]. These approaches can be coupled to number theory, to p-adic numbers. In Bohm’s terminology, we start with pre-space given by the p-adic manifold. This space is considered as a zero dimensional (as a topological space) totally disconnected and disordered space (so, its structure matches with Bohm’s heuristic image of implicated order [12]). It was noted that p-adic numbers have already been widely used in physics, but as a batch of concrete models in string theory, gravity, cosmology, quantum physics, and complex disordered systems [13,14,15,16,17]. Our theory aimed for the reconstruction of the whole body of physics in the very general and consistent framework. The main distinguishing feature of our theory is solid coupling with experimental data. From the data, the implicate order behind such data could be reconstructed. The latter resolves the basic mysteries of modern physics by explaining the origin of nonlocal quantum correlations and collapse of the wave function in a natural way and provided a solution for the measurement problem. At the same time, when keeping to the realist description of nature, the points of p-adic pre-space can be treated as a kind of hidden variable. However, the transition from the zero-dimensional space of hidden variables to experimental data is not straightforward, and it includes a few steps (with growing dimension of structures at each step).

Thus, starting in the number-theoretical framework [18], we proceed to experimental data. As indicated, the pathway from the implicate to explicate order is not straightforward. p-adic numbers have a tree-like formation (homogeneous tree with p edges leaving each vertex and one incoming edge). The first step toward explicate order is an association with each point of the p-adic space with respect to its expansion into the p-adic series. This process generates a one-dimensional string of digits such that aj = 0, 1,…, p−1 (for p = 2, binary strings), and geometrically, this string is a branch of the p-adic tree. The p-adic tree is the source of huge variety of dendrograms termed finite subtrees. These subtrees belong to the domain of explicate order. These subtrees are elementary structures of our theory (explicate order layer), and they play the role of elementary particles in quantum physics. It can be noted that these are two-dimensional structures. To describe interactions between dendrograms, a four-dimensional space is needed. Thus, our theory immediately determines the four-dimensional structure of physical processes. This way of representation of physical processes can be considered a special realization of the holographic principle in the very special form in which the zero-dimensional p-adic horizon encodes dynamics in three-dimensional space through their dendrogram representation, a dendrogramic hologram.

Dendrograms can have different degree of complexity, and simpler dendrograms have higher degrees of correlation. Correlations are generated because of the repeatability of dendrograms of low complexity and not as expressions of causality. At this stage, calculation of correlations, dendrograms partially lose their individuality and are treated as a partly indistinguishable structures. In our approach, indistinguishability is quantified, and it is not just a simple yes/no but up to some degree (which corresponds to all possible combinations of dendrogram structures that can be constructed from N edges). Higher degrees of indistinguishability (or more accurately smaller size of phase space of all possible dendrograms constructed from N edges produces higher indistinguishability) can generate stronger correlations. Bell correlations are modeled in this way as correlation of indistinguishable dendrograms.

Planck’s constant can be coupled to this degree of indistinguishability. From this viewpoint, Planck’s constant can be introduced not only in quantum physics but in any sufficiently complex system as characteristics of indistinguishability between its subsystems (not only in physics, but also biology, economics, and social science).

We finally comment on coupling to Bohm–Hiley’s [8] unification of mental and physical processes based on implicate–explicate orders. The neural networks of the brain have a treelike organization. In the simplest ideal model, the brain can be represented by p-adic numbers. Conscious information processing (explicate order) is described by the quantum potential on pre-space [19] (see the Appendix A for the further discussion). This theoretical construction led to applications to medicine through dendrogram [20] representation of brain electroencephalographic (EEG)-signals and reconstruction of the Bohmian quantum potential.

## 2. Reality Is Obscured by the Assumption of Ergodicity

We now introduce the foundational principle of our theory, the Leibniz principle (hereafter called the Principle):

The identity of indiscernibles is usually formulated in a specific manner such that if, for every property F, object x has F if and only if object y has F, then x is identical to y, or in the notation of symbolic logic: Ɐ F(Fx ↔ Fy) → x = y, namely: if x and y are distinct, at least one property that x has and y does not or vice versa can be found.

Ergodicity is an assumption that was introduced by statistical mechanics in order to understand the “Universe” macro-state from “undistinguished” or partially “undistinguished” collections of micro-states. This assumption of “indistinguishability” of micro-states is clearly wrong and was originally formed for the construction of an adequate model to describe the “Universe” with minimal understanding of the micro-states [21] (see also Martineau et al. for a general discussion on coupling of ergodicity and indistinguishability [22]).

As we see, ergodicity is just a simplification of reality (or rather the consequence of intentionally using Leibniz principle to make properties of the micro-states indistinguishable) in which it is known that each micro-state in the universe has at least one attribute different from the other at a given time (space and time coordinates, momentum, angular momentum, energy, spin, among others). Thus, we have to reject this simplification/assumption. Otherwise, a very ambiguous way is used to describe nature, for example, at our convenience an apple on a tree will be identical to an apple on the ground based on features that ignore space and time (or a tennis ball will be identical to a planet based only on the scale-free geometrical features), but in another feature choice, they will be non-identical or if we simplify and “turn our head” from distinguishable features of two micro-states we face the non-realist consequence of Leibniz principle that an “apple” on a tree is the same “apple” on the ground and is indistinguishable (or we can make any X indistinguishable from Y even if X is a planet and Y is a tennis ball).In this sense, Bell’s experiment is flawed by the simplification assumption that we consider all photons indistinguishable in all properties but its spin and all same spin photons are completely indistinguishable. Moreover, the application of ergodicity in science is flawed twice, not just in the simplification process. Basically, the process in science proceeds in the following manner:

The three-step process (TSP) is as follows:

(1) Detachment from contextuality all micro-states (in a more rigorous manner, a microstate from all or significant portion of its relations to the “universe” is isolated).
(2) Using the above process is assumed that the microstates are indistinguishable.
(3) It is attempted to insert these indistinguishable micro-states back into the “Universe” in which they were before step 1 became clearly distinguishable.

So, in fact a different “Universe” exists in which a “universe” of distinguishable micro-states existed before the three steps. After the three steps, a “universe” of indistinguishable micro-states exists. These three steps are applied to quantum/statistical physics, economical models (indistinguishability of buyers/sellers), social science, and biological and medical sciences.

Regarding a very complex system (the “Universe”), if these three steps are followed, correlations are immediately introduced between the sub-systems. These correlations are “side effects” of our assumption of indistinguishability and its immediate consequence of ergodicity.

*When examining the correlation between the two sets of 2-adic values on the right, it can be seen that the correlations did not violate the Clauser–Horne–Shimony–Holt (CHSH) inequality. The same raw data, divided into groups of five datapoints and then clustered into a five-edge dendrogram produced pairs of 2-adic values that violate CHSH inequality.*

## 3. Correlation Emergence: Methods and Results

As an example, we reproduced the Bell violations of correlations from a very classical double slit diffraction experiment was reproduced [23]. The original experiment used a CCD camera with 512 × 512 detectors chip. The detection pattern in each frame in the experiment was represented as a binary matrix of 512 × 512 were 0 values represent no detection of photons in the corresponding detector while 1 value represent detection of photons in the corresponding detector for each frame and its corresponding binary matrix we found all column positions that had the value 1, multiplied these positions value and calculated the log_10_ value of the outcome, this resulted in a unique number representing each frame detection pattern. Thus, each frame was encoded with only one unique number $A_{f}$ in which:

(1) f∈frame 1 2...n

From each number of consecutive frames, N, the pairwise distances between each of the $N\;A_{f}$ was calculated and a dendrogram using ward linkage was constructed (*Ward’s linkage* uses the incremental sum of squares, that is, the increase in the total within-cluster sum of squares as a result of joining two clusters. The within-cluster sum of squares is defined as the sum of the squares of the distances between all objects in the cluster and the centroid of the cluster. The sum of squares metric is equivalent to the following distance metric *d*(*r*,*s*)). Here we follow the works of Murtagh et al. [24,25,26] devoted to hierarchical clustering of data and its coupling to ultrametric and p-adic models.

Each dendrogram was then represented in a matrix in which each row (*r*) represented the 2-adic expansion of the edge route in the dendrogram tree. Each 2-adic expansion was converted to a rational number using the equation:

(2) $$q=\sum^{\;}2^{-find\left(r=1\right)}\;,\;q\;\in \;\left[01\right]$$

**Figure 1:**
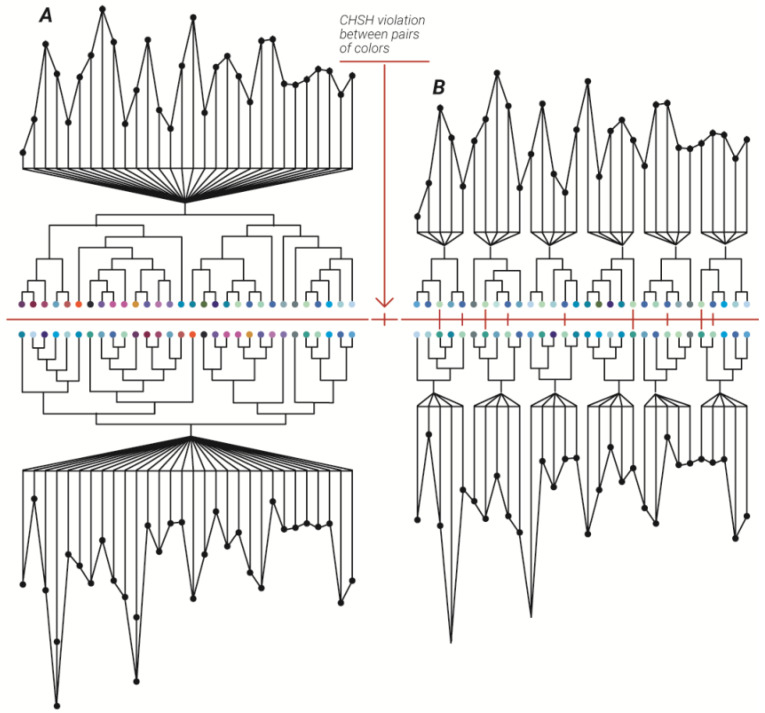
The emergence of quantum correlations. Illustration of the three steps process (TSP) leading to quantum correlations. Two sets of raw data (black dots) are clustered into two dendrograms each containing 30 edges (**A**). The same data were clustered into two sets of six dendrograms each with five edges (**B**). Colored spheres represent the 2-adic values of an edge route in the dendrogram.

**Figure 2:**
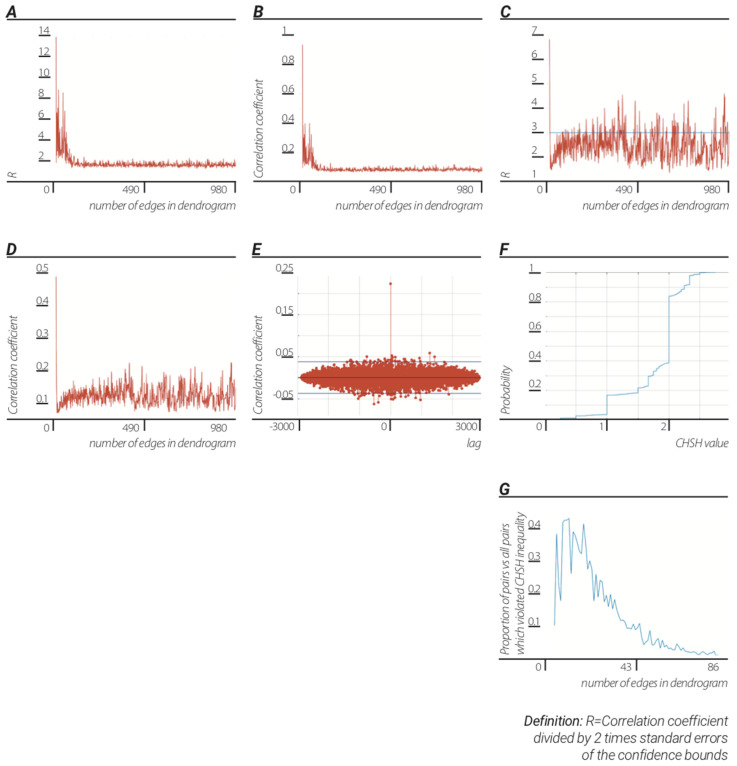
Quantum correlations from classical data and the transition from quantum to classicality. The emergence of quantum correlations and transition to classicality is a. consequence of the TSP: (**A**) significance of correlation between two sets of quantum potential values versus number of edges in unit dendrogram. Values were calculated for each unit dendrogram with fixed number of edges (N = 3, 4, 5…980) after which two temporal halves of quantum potential values were then cross-correlated. (**B**) correlation coefficients of the two sets of quantum potential values versus number of edges in unit dendrogram. (**C**) significance of correlation between two sets of p-adic values versus number of edges in unit dendrogram. Values were calculated for each edge route in unit dendrogram (as described in Section 3). (**D**) correlation coefficients of the two sets of p-adic values versus number of edges in a unit dendrogram. (**E**) correlation between detectors of real bell experiment. (**F**) fraction of CHSH values versus CHSH values. CHSH values were calculated for each possible pair of p-adic values obtained from two halves of the diffraction data divided by unit dendrograms of size N = 5…86. (**G**) The proportion of p-adic pairs that produce CHSH values which violate the CHSH inequality versus number of edges in a unit dendrogram.

Following, for each edge *i*, i∈1,2...N, in which N is the number of edges in dendrogram, *q_i_* (this corresponds to step 1 and 2 in the three steps process described in Section 1 as each where the unit dendrogram with N edges, each edge represents one unique $A_{f}$ number which in turn represents one frame of the original experiment, is our very simple electron, photon, among others) was calculated and then repeated for the next N, $A_{f}$, unique numbers (Figure 1). We next proceeded to step three in the TSP described in Section 1 by inserting these unit dendrograms back into the “Universe” in a chronological manner. The first half of these q 2-adic values were then correlated after which they were correlated with the second half. The mean and standard deviation (std) of the ratio of correlation coefficient to 2-times the standard error (SE) of the confidence bound for N edges unit dendrogram (N = 3, 4…980) were 2.4884±0.5775 (Figure 2c). The mean and std of the correlation coefficient for N edges unit dendrogram (N = 3, 4…980) were 0.1243±0.0299 (Figure 2d; first temporal half of the diffraction frames correlated to the second temporal half of the diffraction frames).

Moreover, the p-adic quantum potential field of each such dendrogram was calculated and for each edge, its quantum potential value was calculated in the following manner: In order to extract the 2-adic quantum potential double slit diffraction experiment, the estimated probability distribution function (pdf) *ρ(q)* of $q_{i}$ with a kernel function of bandwidth was constructed:

(3) $$\;(\max\left(q_{i}\right)-\min\left(q_{i}\right))/N$$

The quantum potential field (*QP*) was calculated according to Holland [27] as shown below:

(4) $$QP=\frac{1}{4\rho }\left(\frac{1}{2\rho }\frac{\partial \rho }{\partial q}\frac{\partial \rho }{\partial q}-\frac{\partial {\;}^{2}\rho }{\partial q\partial q}\right)$$

were ρ is the distribution function of p-adic values q.

The quantum potential in this formulation is very trivial information measure of the dendrogram topology. Each edge was attributed with its QP value. QP values of the first half of the diffraction experiment to the second half figures were correlated. The mean and std of the correlation coefficient ratio to std of the cross correlation sample and of the of the correlation coefficient for N edges unit dendrogram (N = 3...980) were 1.8079±0.7727 and 0.0923±0.0420, respectively (Figure 2a,b; first temporal half of the diffraction frames correlated to the second temporal half of the diffraction frames). Both analyses described above resulted in very good correlation when compared to the correlations with the detectors of a real bell experiment (Figure 2e).

In order to test the Bell violations present in the dendrogramic representation, the Clauser–Horne–Shimony–Holt (CHSH) inequality violations for each pair of the unique p-adic values (or rather p-adic features) that were produced from the two halves of the diffraction experiment upon the above-described transformation to dendrogramic units.

When reviewing at all data representations with unit dendrograms of increasing number of edges (N = 5, 6,…86) combined, we found that although most (~80% of the unique p-adic pairs) showed classical correlations, ~20% of the unique p-adic pairs showed violation of bell inequality (Figure 2f). With increasing N of dendrogram edges, the fraction of frames in the diffraction experiment (represented as p-adic numbers) that violated the CHSH inequality decreased. Thus, upon dividing the data to very simple unit dendrograms (small number of edges) very well mixed quantum and classical correlation was produced. Progressively, when the data are divided into more complex unit dendrograms (high numbers of edges), a reduction of quantum correlations and the increase of classical correlations can be observed. Thus our model implies a mechanism for the transition from the quantum to the classical domain (Figure 2g).

We note that the transition to the classical domain was a clear indication of the finite “universal” dendrogram, and upon reaching it, no quantum correlations were present as each edge was uniquely determined and different from others. Thus, this analysis supports a finite explicate universe within a bounded implicate universe. This paradigm was partially advocated by Bohm [12], Smolin [28], and Barbour [29].

Our physical theory is supported by extended mathematical modelling of the process of percolation [30]. Percolation is concerned with the study of random subgraphs of a given graph (G) and more specially, its infinite connected components, which are called clusters. The fundamental theorem of Lyons and Schramm [31] states that, under some constraints, if several infinite clusters are produced, they all “look alike”. the rigorous formulation of this theorem is presented in this study without going into a deeper explanation of mathematical notions related to it:

**Theorem** **1.** *(Cluster indistinguishability): Let G be a graph with a transitive unimodular closed automorphism group **g**, a subgroup of Aut(G). Every **g**-invariant, insertion-tolerant, bond percolation process on G has indistinguishable infinite clusters.*

In conclusion, by reproducing Bell correlations from a “classical system” we demonstrated that the emergent nonlocality was a side effect of the assumption of indistinguishability/partial indistinguishability. Moreover, the transition from the “quantum domain” of very small phase space of simple dendrograms to the “classical domain” of more complex and larger phase space of complex dendrogram has been demonstrated (Figure 2G).

## 4. Planck’s Constant Meaning

We claim that Planck’s constant is actually a measure of the indistinguishability of sub-systems (from photons → electrons → atoms → heat baths), so Planck’s constant will emerge for any system that is divided into indistinguishable/partly indistinguishable sub-systems.

## 5. Reflections on Relation to Holographic Principle and Horizon

**Figure 3:**
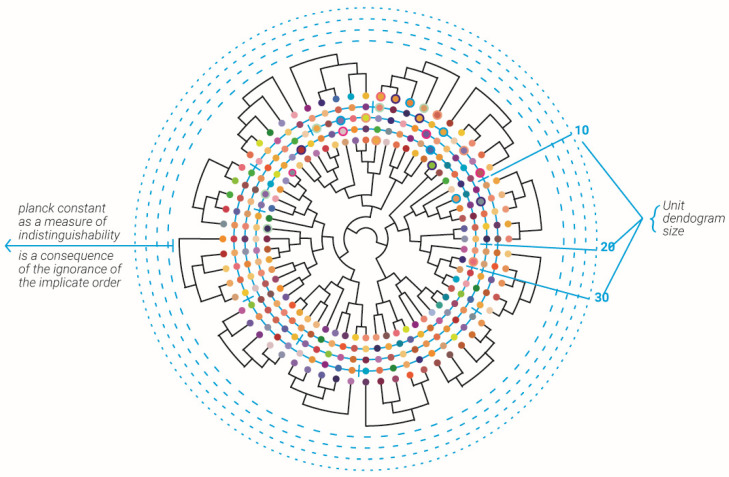
Abstract description of the implicate and extrinsic order alongside the emergence of Planck’s constant as a measure of indistinguishability. The “Universal” dendrogram is shown at the center of the circle with its color spheres edges describing each edge 2-adic expansion values of edge route. The outer spheres circles describe the 2-adic values of each edge in the “unit” temporal dendrogram with decreasing edge numbers N = 20, 10, 5. Each unit dendrogram 2-adic values were best-matched to its corresponding edges in the smaller (higher edge number) circle compared to its non-corresponding edges (see for example 5 spheres with rings in each circle). Planck’s constant is the measure of indistinguishability, which is a consequence of the ignorance of each dendrogram edge from the infinite sub dendrograms that continue from it (outer dotted circles).

Let the whole “Universe” be represented as a giant dendrogram in which all of the information in that universe is present at the circumference with no dynamics, and inside the circle, the dynamics of information emerges (Figure 3). Now, it was suggested that Planck’s constant represents a fixed amount of information bits [32,33,34]. This complex dendrogram with all information is contained in the edge’s routes at which the edges are on the horizon (of the dendrogram). When coarse grain edges of the dendrogram are started or rather used in only a disconnected fashion, detached sub-dendrograms of the complex “Universal” dendrogram dynamics emerge.

**Figure 4:**
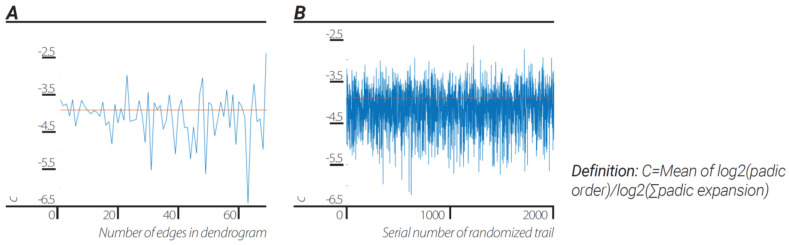
**Emergence of Planck’s constant and speed of light**. Ratio of log2 (p-adic order of edge) to log2 (sum of edge p-adic-expansion) corresponded to the ratio by dividing the data to fixed number of edges dendrograms (**A**) and by dividing the data to a dendrograms with random number of edges (**B**).

**Results.**  *The universal dendrogram was coarsely grained by joining fixed number (Figure 4a)/random number of edges (Figure 4b). The ratios between “p-adic order of edges”/”sum of p-adic-expansion” was* −4.0831±0.6331*and*−4.1677±0.5071 *for fixed number of edges and random number of edges, respectively, which was approximately the ratio of:*

(5) $$\frac{log2\left(h\right)}{log2\left(c\right)}\approx -3.91$$

Please note that at relatively small N (N = 5, 6...15) compared to the number of edges in the “Universal” dendrogram, the mean result was in agreement up to 0.6% of the log2(h)/log2(c) ratio (Figure 4a).

## 6. Implicate Order Present in the Explicit Order

**Figure 5:**
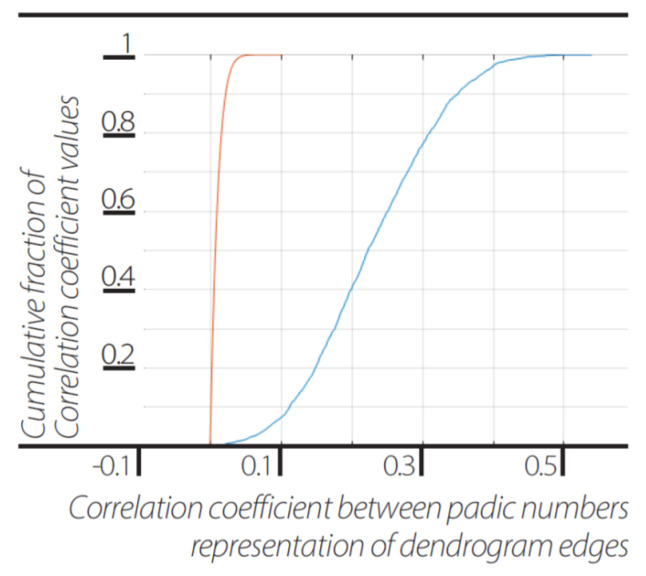
**Implicate order encoded in explicate order**. Cumulative distribution function (cdf) of correlation between the same events represented as 2-adic values in dendrograms of different sizes N = 5...3920 (blue line) and cdf of the correlation between different events represented as 2-adic values in dendrograms of different sizes N = 5…3920 (orange line).

Upon correlating the unit simple dendrogram (or its p-adic numbers representative of its edges) in terms of cross-correlation coefficient, it was found to be best matched to all of its corresponding components in the “Universal” dendrogram or to its corresponding components in the more complex and larger dendrograms (0.2281±0.0910) than if it was compared to the dendrogram’s non-corresponding edges in the universal dendrogram or non-corresponding edges in the more complex dendrogram (0.0099±0.0088). Hence, the holographic principle in terms of implicate and explicate orders by Bohm [12] was satisfied (Figure 5).

## 7. Wave Function out of Classical Subsystems

Suppose a very large “Universe” of heat bath exists. Sufficiently equal sized large units of sub- heat baths are detached. Each heat bath state can be represented by a finite dendrogram. It can be claimed that if the sub-heat baths have the same N of particles, a finite set of dendrograms that can represent all of them and all of their possible states should exist. This finite set may be considered a “wave function” and upon measuring one particular unit of sub-heat bath, the structure of its dendrogram can be realized, and thus the collapse of the “wave function” will occur. Notice, as already shown in Section 6, that each sub-heat bath dendrogram was very well correlated to its corresponding edges in the dendrogram of the “universal” heat bath.

## 8. Measurement Process as Dynamical Process in our (Based on Lee Smolin’s and Julian Barbour’s) Maximal Variety Dynamics

Consider an ensemble of dendrograms. As was shown (Section 6), each unique dendrogram in the ensemble correlated best to its corresponding edges in the “universal” dendrogram or in the more complex dendrograms containing these edges.

**Figure 6:**
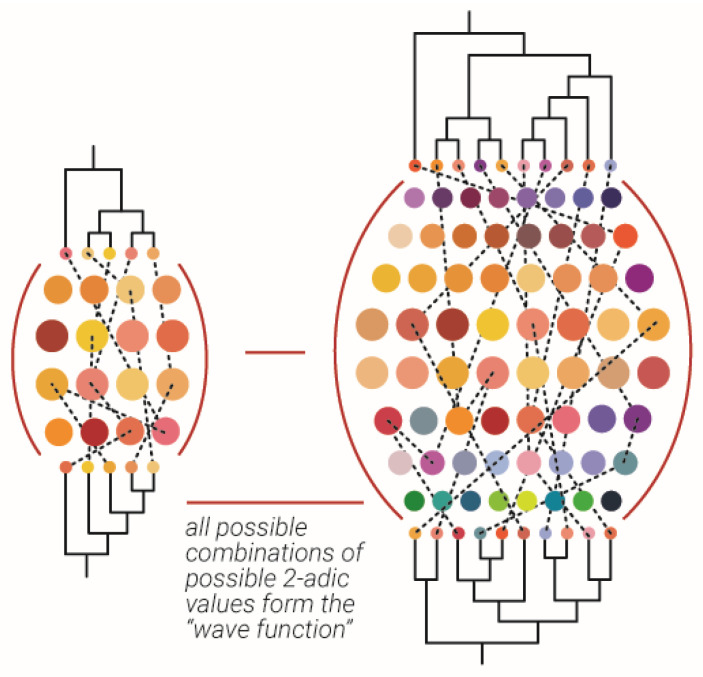
Measurement of the wave function according to maximal variety principle**.** An abstract example of measurement of the wave function which is the ensemble of possible combinations of possible 2-adic values, which is illustrated as a collection of colored spheres between two dendrograms. The maximal variety principle suggest both dendrogram on each side of the “wave function” should be maximally different.

Thus, when one unique dendrogram in the ensemble interacts with the ensemble, it will measure a maximal different unique dendrogram (such as in the maximal variety principle); in doing so, this unique dendrogram will provide much more information on the structure of the whole “universal” dendrogram (Figure 6).

**Figure 7:**
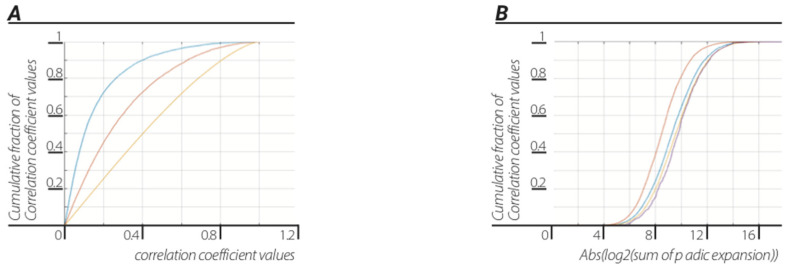
**Wave function measurement and emergent dynamics from maximal variety principle.** CDF of correlation coefficients in the diffraction experiment between temporally ordered 2-adic representation of edges of dendrograms of different sizes N = 5...980 (**A**, blue trace). CDF of correlation coefficients in the diffraction experiment between non-temporally ordered 2-adic representation of edges of dendrograms of different sizes N = 5...980 (**A**, orange trace). CDF of correlation coefficients between randomly constructed, 2-adic representation of edges of dendrograms of different sizes N = 5...980 (**A**, yellow trace). Coarse graining the universal dendrogram for each N = 5...980 frames show differences in p-adic representation between consecutive chronological dendrograms coarse grained edges (**B**, blue trace) non chronological dendrograms coarse grained edges (**B**, orange trace) randomization of chronological sequence of dendrogram edges before coarse graining (**B**, yellow trace), random binary data (**B**, purple trace).

The dynamical change in our “Unit” dendrograms was quantified by cross-correlating their p-adic representation first (Figure 7a, blue trace) to the “chronological” consecutive and next with median of the cross-correlation coefficients (0.1016). It was then cross-correlated (Figure 7a, orange trace) between all non-chronological unit dendrograms, with median of the cross-correlation coefficients 0.2300, thirdly (Figure 7a, yellow trace) two pairs of random sets of N p-adic values (N as the number of edges in the unit dendrogram and each p-adic value is one value out of all possible of $2^{\left(\mathrm{maximal}\;\mathrm{branch}\;\mathrm{length}\;\mathrm{of}\;\mathrm{actual}\;\mathrm{unit}\;\mathrm{dendrogram}\right)}\;$combinations) were correlated to each other, with median of the cross-correlation coefficients 0.4025. For all different sizes of unit dendrograms the results are shown (Figure 7a).

The chronological unit dendrograms “chose” to interact with much different other, much different unit dendrograms (Figure 7a; blue), while the non-chronological cross-correlation between “unit” dendrogram was much more similar (orange). Moreover, random dendrograms with same edge numbers were even more similar (yellow). This finding is in accordance to the maximal variety principle [9,35] alongside the proposition made in Section 7 on wave function of heat bath ensembles and the collapse of such wave function upon measurement.

When the universal dendrogram for each N frames in the “Universal” as one edge in a new dendrogram was coarse grained, it can be seen again that a clear distinction between differences in p-adic representation between consecutive chronological dendrograms compared to the p-adic representation of non-chronological dendrograms edges were found (Figure 7b). Moreover, the coarse-grained chronological p-adic representation differences resembled randomized data with median values for non-chronological of 8.4681, chronological of 9.2804, randomized data 9.5731, and random binary data of 9.7620.

In conclusion, we demonstrated that the measurement process between two dendrograms occurs between two maximally different topologies in order for both topologies to experience different locations of the universal dendrogram; this process is in accordance with Section 6 in which the connection of the implicate order to the explicit order was shown. In more detail, a sub-system in the explicit order was best-fitted to itself in the implicate order (more complex dendrogram). Thus, as our results in this section show, only two very different sub-systems would interact/measure with each other, which in turn reflected interactions between two very different areas of the implicate order. We emphasize that the measurement process is in fact dynamic (more accurately, apparently dynamic). These statements are well supported with our analysis in this section and Section 6 above and in accordance with Roveli’s (and Smolin’s) relational quantum mechanics interpretation [36]. Thus we modeled the measurement process as a linkage/relational property of two sub-systems in which, in fact, the apparent dynamics unfolds/reveals different areas of the “universal” dendrogram and the implicate order. Moreover, by using empirical data, the validity of the maximal variety principle suggested in past studies was confirmed [35,37].

## 9. Reconstruction of Space Time Using the Maximal Variety Least Action Principle

In accordance with recent studies [6,38], the least action principle, which is based on the maximal variety principle [9], is a very natural consequence of our analysis. Thus, how well the chronological “Universal” dendrogram (edge by edge according to real chronological sequence) fits with the chronological time and position of detectors in the diffraction experiment that detected photons in each frame was examined.

The measure of distinguishability between all edges was encoded in the dendrogram and defined:

(6) $$V_{i}=2-adic\;point\;value\;of\;edge\;in\;dendrogram$$

is the measure of distinguishability of edge *i* from all edges j≠i.

The potential energy was taken to be proportional to the negative of the measure of distinguishability or variety in Lee Smolin’s and Julian Barbour’s terminology [6,37,38]:

(7) SRE=gV

$$q_{ij}=\;momentum\;from\;V_{i}\;to\;V_{j}\;i,j\in 1\ldots n=number\;of\;edges\;in\;dendrogram\;,\;i\neq j\;$$

Is a measure of distinguishability between edges i and j.

The energy momentum relationships are shown below:

(8) $$\frac{q_{ij}^{2}}{2}=E_{kinetic}$$

As we showed in Section 8, $q_{ij}$ should be maximal through the chronological measurement/dynamic process. Thus:

(9) $$P_{i}=q_{ij}-q_{jk}\;tend\;to\;be\;0\;in\;order\;that\;\overset{n}{\underset{i=1\;j=i+1}{\sum }}q_{ij}$$

will be maximal, and our version of the action is slightly different than in the above mentioned study [6]:

(10) $$S^{ECS}+S^{RE}=\overset{n}{\underset{i=1\;j=i+1}{\sum }}\tilde{N}\frac{q_{ij}^{2}}{2}+\sum_{i=1}^{n}{\tilde{z}}_{i}P_{i}+gV$$

The variation by $q_{ij}$ yielded:

(11) $$0=\tilde{N}q_{ij}+{\tilde{z}}_{i\;}+g\frac{\partial V_{i}}{\partial q_{ij}}$$

**Figure 8:**
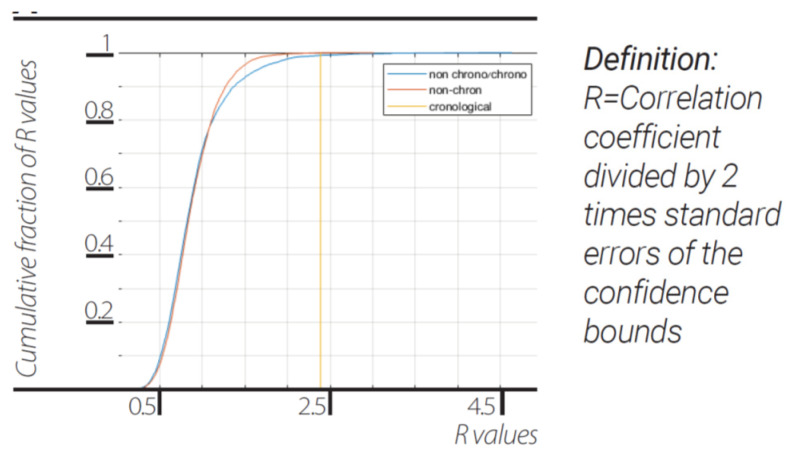
Reconstruction of space-time intervals. Correlation coefficient divided by 2-times the standard errors (SE) of the confidence boundaries of reconstructed spacetime intervals from randomized walk on full dendrogram edges sequence correlated to its randomized real space time intervals (blue trace). cdf of correlation coefficiants of reconstructed spacetime intervals from randomized walk on full dendrogram edges sequence correlated to real chronological spacetime intervals (orange trace). Correlation coefficient value of the real chronological spacetime intervals correlated to the reconstructed intervals from chronological walk on full dendrogram edges (yellow vertical trace).

As shown previously [6], the spacetime intervals were represented by the Lagrange multiplier ${\tilde{z}}_{i}$ after substituting $q_{ij}$ and $V_{i}$ into the equation above. We calculated ${\tilde{z}}_{i}\;$and correlated the result with real space and time in the diffraction experiment data [23] (Figure 8). We evaluated the values of the correlation coefficients divided by 2 times the standard error of the confidence bound, which yielded the value of the real chronological spacetime intervals. These values were correlated to the reconstructed intervals is in the upper 0.9922 and 0.9998 percentile of the values for randomized walk on dendrogram edges sequence correlated to the real chronological spacetime and to the real, but non-chronological, spacetime, respectively.

## 10. Differences between Our and Smolin’s Theories

In our model, kinetic energy and momentum are consequences of measurement (that produce an apparent dynamic) between unit and unit dendrograms or edge and edge in the universal dendrogram ($q_{ij}$).

Potential energy (V) is defined as a measure of distinguishability, which is encoded in the Universal dendrogram or in the coarse-grained dendrogram.

The quantum potential encodes the information contained in the topology of the dendrogram. It is possible to compute the quantum potential for unit dendrogram or for the universal dendrogram.

In contrast to Smolin [1,2,6,37], we emphasize that all p-adic points are always present (this emphasis is more in accordance to Barbour’s [29,35] “always present events’’) and do not need to appear by a dynamic process. In contrast to Barbour, we did not require probabilities in phase space to produce the apparent dynamics.

We note that we do not need to postulate the fundamentality of time momentum and energy as in Smolin’s study [6,37,38].

We note again that at the implicate order level, all zero dimensional points of the Universe already exist and are not dependent on referenced measurement (as the holographic principle clearly states for the horizon). Only upon the process of expansion into p-adic expansion strings does the p-adic points acquire a relational nature among themselves. So, in fact we grew from nonrelational dimensionless p-adic numbers (points) to relational information between p-adic expansions (encoded as one-dimensional information string) to dendrograms (as two-dimensional information structure) and then to dynamics (relationships) between points/dendrograms (coarse grained) that produce spacetime. It should be noted that the dimensionality of spacetime is produced by the sequence 2^-∞ → 2^0 → 2^1 → 2^2. Thus, although our theory is relational, and at its core, it is a collection of non-referenced determined numbers and thus a purely number theory.

## 11. Concluding Remarks

The presented DH-theory is an attempt to re-establish realism in physics (including quantum mechanics) by combining it with so popular nowadays information approach to physics starting with Wheeler’s [39] “it from bit” to modern quantum information reconstructions of quantum theory, such as found in a study by D’Ariano [40]. We formalized the Bohmian implicate versus explicate order structuring of physics via p-adic number theoretic versus dendrogram representations. The latter is of the purely information nature. All observable physical processes are realized as interactions between “universal’’ dendrograms representing physical systems in which the “Universe” is modeled as a huge dendrogram; four-dimensional spacetime physics is reconstructed based on the holographic principle. Classical-quantum interplay was quantified through the degree of indistinguishability between dendrograms. Dendrograms can be considered special mathematical structures in Smolin’s views on events. His least action principle was used to connect dendrogramic model with processes in physical space-time.

We restricted matching of our theory with the real experiment to the data from diffraction experiment but in fact it can be applied to any classical or quantum. In the future, we plan to perform extended analysis of the experimental data both from quantum physics and statistical mechanics to validate further data matching with our theory.

Finally, we remark that creation of the DH-theory can be considered as the important step in coupling the mathematical models developed within p-adic theoretical physics [14,15,16,17,18] with real experimental data and correlations produced in classical and quantum physics. We remark that p-adic theoretical physics was born within string theory. Heuristically theory of interacting dendrograms has some similarity with theory of interacting strings. We also point to the monograph [19] in that the basic model with p-adic pre-space was presented in very detail although without any coupling to real experimental data (or even the method for such coupling).

## Data Availability

Not applicable.

## Appendix A. p-Adic Quantum-Like Models, Cognitive Applications and Possible Generalizations

We remark that creation of the DH-theory can be considered as the important step in coupling the mathematical models developed within p-adic theoretical physics [14,15,16,17,18,41,42,43] with real experimental data and correlations produced in classical and quantum physics. We remark that p-adic theoretical physics was born within string theory. Heuristically theory of interacting dendrograms has some similarity with theory of interacting strings. We also point to the monograph [19] in that the basic model with p-adic pre-space was presented in very detail although without any coupling to real experimental data (or even the method for such coupling). This monograph also presents the p-adic quantum-like approach to modelling of cognitive processes in the brain (see also [44,45]). Quantum-like means that the brain is considered as a black box (macroscopic) processing information in accordance with the laws of quantum theory. Quantum-like modelling of cognition has to be sharply distinguished from genuine quantum physical modelling in the spirit of Penrose and Hameroff [46,47]. The quantum-like p-adic model of brain’s functioning [19] was applied for medical diagnostic of psychic disorders [20]. It seems that this is the first application of p-adics as well as quantum-like modelling to medicine. The use of p-adic quantum potential reconstructed from patients’ EEG time series provides the novel and very powerful method of diagnostics.

p-adic trees are homogeneous and they provide the simplest model of implicate order. The model can be extended by consideration of inhomogeneous trees and corresponding ultrametric spaces. Regarding the algebraic structure, the model can be reformulated by relying on a generic absolutely valued division ring whose endowed topology is zero-dimensional and totally disconnected, and which does not admit a total order compatible with its ring structure. These features match well Bohm’s image of implicate order.
